# Supplementary material for: Participation of the ABC Transporter CDR1 in Azole Resistance of Candida lusitaniae
Source: J Fungi (Basel). 2021 Sep 15;7(9):760. doi: 10.3390/jof7090760 (PMC8467326; doi:10.3390/jof7090760)
Supplement: Supplementary file 1 [file jof-07-00760-s001.zip › Fig S1.pdf]

Fluconazole

	P1	P1-*mfs7* Δ	P1-*cdr1* Δ	P1-*mrr1* Δ	P1-*mfs7* Δ *cdr1* Δ	P1-*mrr1* Δ *cdr1* Δ
**P1**	0	-1	-1	1	-2	1
**P1-*mfs7* Δ**	1	0	0	2	-1	2
**P1-*cdr1* Δ**	1	0	0	2	-1	2
**P1-*mrr1* Δ**	-1	-2	-2	0	-3	0
**P1-*mfs7* Δ *cdr1* Δ**	2	1	1	3	0	3
**P1-*mrr1* Δ *cdr1* Δ**	-1	-2	-2	0	-3	0

Posaconazole

	P1	P1-*mfs7* Δ	P1-*cdr1* Δ	P1-*mrr1* Δ	P1-*mfs7* Δ *cdr1* Δ	P1-*mrr1* Δ *cdr1* Δ
**P1**	0	-1	0	-1	-1	-1
**P1-*mfs7* Δ**	1	0	1	0	0	0
**P1-*cdr1* Δ**	0	-1	0	-1	-1	-1
**P1-*mrr1* Δ**	1	0	1	0	0	0
**P1-*mfs7* Δ *cdr1* Δ**	1	0	1	0	0	0
**P1-*mrr1* Δ *cdr1* Δ**	1	0	1	0	0	0

Itraconazole

	P1	P1-*mfs7* Δ	P1-*cdr1* Δ	P1-*mrr1* Δ	P1-*mfs7* Δ *cdr1* Δ	P1-*mrr1* Δ *cdr1* Δ
**P1**	0	0	0	0	-1	-3
**P1-*mfs7* Δ**	0	0	0	0	-1	-3
**P1-*cdr1* Δ**	0	0	0	0	-1	-3
**P1-*mrr1* Δ**	0	0	0	0	-1	-3
**P1-*mfs7* Δ *cdr1* Δ**	1	1	1	1	0	-2
**P1-*mrr1* Δ *cdr1* Δ**	3	3	3	3	2	0

Max

Centile

Min

Figure S1: Pairwise comparisons of MICs between isolate P1 and derived mutants. Fold-changes are given in log2 scale. Comparisons were made always starting from top row. Values > 0 indicate MIC decreases, while values < 0 indicate MIC increases. Greyed colored boxes indicate comparisons between same isolates.
