# Supplementary material for: Participation of the ABC Transporter CDR1 in Azole Resistance of Candida lusitaniae
Source: J Fungi (Basel). 2021 Sep 15;7(9):760. doi: 10.3390/jof7090760 (PMC8467326; doi:10.3390/jof7090760)
Supplement: Supplementary file 1 [file jof-07-00760-s001.zip › Supplementary Tables.pdf]

Table S1: list of strains used in this study

Strain	Name in this study	Parental strain	Genotype	Reference
DSY4606	P1	-	Wild type	[1]
DSY4593	P3	DSY4606	*MRR1*^{V668G}	[1]
DSY5416	P3 *mrr1*Δ	DSY5241	*mrr1*Δ::FRT	[2]
DSY5437	P3 *mrr1*Δ + *MRR1*	DSY5416	*MRR1*::*NAT1*	[2]
DSY5439	P3 *mrr1*Δ + *MRR1* ^{V668G}	DSY5416	*MRR1* ^{V668G}::*NAT1*	[2]
DSY5639	P3 *mrr1*Δ + *MRR1* ^{G654A}	DSY5416	*MRR1* ^{G654A}::*NAT1*	This study
DSY5247	P3-*mfs7*Δ	DSY4593	*mfs7*Δ::*NAT1*	[2]
DSY5248	P1-*mfs7*Δ	DSY4606	*mfs7*Δ::*NAT1*	[2]
DSY5241	P3-*mrr1*Δ	DSY4593	*mrr1*Δ::*NAT1*	[2]
DSY5658	P1-*mrr1*Δ	DSY4606	*mrr1*Δ::*NAT1*	[2]
DSY5635	P1-*cdr1*Δ	DSY4606	*cdr1*Δ::*HygR*	This study
DSY5607	P3-*cdr1*Δ	DSY4593	*cdr1*Δ::*HygR*	This study
DSY5609	P3-*mfs7*Δ*cdr1*Δ	DSY5247	*mfs7*Δ::*NAT1*/*cdr1*Δ::*HygR*	This study
DSY5611	P1-*mfs7*Δ*cdr1*Δ	DSY5248	*mfs7*Δ::*NAT1*/*cdr1*Δ::*HygR*	This study
DSY5613	P3-*mrr1*Δ*cdr1*Δ	DSY5241	*mfs7*Δ::*NAT1*/*cdr1*Δ::*HygR*	This study
DSY5664	P1-*mrr1*Δ*cdr1*Δ	DSY5242	*mfs7*Δ::*NAT1*/*cdr1*Δ::*HygR*	This study
DSY5643	*MRR1*-*cdr1*Δ	DSY5437	*MRR1*::*NAT1*/*cdr1*Δ::*HygR*	This study
DSY5645	*MRR1* ^{V668G}-*cdr1*Δ	DSY5439	*MRR1* ^{V668G}::*NAT1*/ *cdr1*Δ::*HygR*	This study
DSY5644	*MRR1* ^{G654A}-*cdr1*Δ	DSY5639	*MRR1* ^{G654A}::*NAT1*/ *cdr1*Δ::*HygR*	This study

Table S2: primers used in this study

Primer	Sequence (5' to 3')
ClCDR1-P1	AGGATGAATGGGTTAGCCAAATCG
ClCDR1-TEF-5R	ATTTATTCCGAGCGCTATACAGTATAGTGCTTGCTGTTCGAT
ClCDR1-P2	GACGGCAATGGGAATCAAAATGC
ClCDR1-ACT1-3f	ATTCATCCCATTCATTCCATCGTAATACCCCGCAAAGACCTT
CLCDR1-TEF-5f	ATTTATTCCGAGCGCTATACAGTATAGTGCTTGCTGTTCGAT
ClCDR1-ACT1-3r	AAGGTCTTTGCGGGGTATTACGATGGAATGAATGGGATGAAT
ClCDR1-P3	CACACATTCCATTGGAGGCTCTT
ClCDR1-P4	CAATGTTGCCCCACTCTAAGACCA
Hygro_1949R	ACCATCAGCACAAGAATTAACTCT
ClCDR1-3-verif	GTTGAAGTATTTGGACTCGGAGTT
ACT1-pYM70	CCACCCAAGGCATTTCTATATCTT
pDS1918-P1	CGGCCAGTGAGCGCGCGTAA
V654A-R	AGCTCCCTGTCAATCTGAGCGTCGTAGAAAGTCAAACCCAA
V654A-F	TTGGGTTTGACTTTCTACGACGCTCAGATTGACAGGGAGCT
ClMRR1-SacI	TAGGAAGAGCTCACAATAAGTTTTCATTG
ClMRR1-Apa	TTGTGGGCCCAGAGATCTGTTCTAATGA
MRR1-3_rev_new	GATGAGGATGGCGAGAAGATCAATTCCATCTCCTCTACTCTTGGAACAAAGAGAATTGTCA ATCCTAACTGGTACCCACTTGTTGTTTTCAACGATGACGAGACAGCAGTATAGCGACCAGC
ClMRR1_F	CATTGCACAAAAGCAAAGC
Cl_MRR1_3377_R	TGTGTTGTTCGAAAAGACAGAAAG
ClCDR1_5_Cas9	TGCTTTGTACAAGATGGCGG
ClCDR1_3_Cas9	AAAGACTATATTGCCAAGGC
ClACT1-P [a]	TCTCCTTGCCTCACGGTATCTTG
ClACT1-F	CACGTTGTCCCAATTTAC
ClACT1-R	CTTGCTCAAAGTCCAAAG
MFS7-P [a]	TCTTCATTCCATTCATCTACAGGCGA
MFS7-F	GTTGCTATTATGATTGGTATC
MFS7-R	AGGCATAAGGATAGAACC
ClCDR1-P [a]	TCTATCGGTTATGTCCAGCAGCAA
ClCDR1-F	AACGGTCATGGATTAGAC
ClCDR1-R	CCTTGATACAGAATATGGTTG

[a] : Taqman probes with FAM and TAMRA modifications

Table S3: MICs of clinical isolate DSY4941

Antifungal drugs	MIC (µg/ml)
Anidulafungin	0.12
Micafungin	0.12
Caspofungin	0.12
5-Flucytosine	0.06
Posaconazole	0.12
Voriconazole	0.12
Itraconazole	0.25
Fluconazole	16
Amphotericin B	0.5

Table S4: Antifungal MICs of *C. lusitaniae* isolates and mutant derivatives

Antifungal drugs	MIC (µg/ml)					
	MRR1	*MRR1 cdr1*Δ	*MRR1*^{V688G}	*MRR1*^{V688G} *cdr1*Δ	*MRR1*^{V654A}	*MRR1*^{V654A} *cdr1*Δ
Anidulafungin	0.12	0.12	0.12	0.12	0.25	0.25
Micafungin	0.06	0.06	0.06	0.06	0.12	0.12
Caspofungin	0.25	0.25	0.5	0.5	0.5	0.5
5-Flucytosine	2	4	>64	>64	>64	>64
Posaconazole	0.03	0.015	0.5	0.015	0.5	0.06
Voriconazole	0.008	<0.008	0.25	0.015	0.5	0.25
Itraconazole	0.12	0.03	0.5	0.06	1	0.06
Fluconazole	0.5	0.12	32	1	64	32
Amphotericin B	0.25	0.25	0.5	0.25	0.5	0.5

References

1. Asner, S.A.; Giulieri, S.; Diezi, M.; Marchetti, O.; Sanglard, D. Acquired Multidrug Antifungal Resistance in Candida Lusitaniae during Therapy. *Antimicrobial Agents and Chemotherapy* 2015, *59*, AAC.02204-15-8, doi:10.1128/aac.02204-15.
2. Kannan, A.; Asner, S.A.; Trachsel, E.; Kelly, S.; Parker, J.; Sanglard, D. Comparative Genomics for the Elucidation of Multidrug Resistance in Candida Lusitaniae. *mBio* 2019, *10*, 1227–21, doi:10.1128/mbio.02512-19.
